# Learning the mechanism of collective microbial function via random community-media pairing

**DOI:** 10.1088/1478-3975/aeb3c4

**Published:** 2026-11-11

**Authors:** Luoqi Wang, Seppe Kuehn, Mikhail Tikhonov

**Affiliations:** 1 Graduate Program in Biophysical Sciences, The University of Chicago, Chicago, IL 60637, United States of America; 2 Department of Ecology and Evolution, The University of Chicago, Chicago, IL 60637, United States of America; 3 Center for the Physics of Evolving Systems, The University of Chicago, Chicago, IL 60637, United States of America; 4 Center for Living Systems, The University of Chicago, Chicago, IL 60637, United States of America; 5 National Institute for Theory and Mathematics in Biology, Northwestern University and The University of Chicago, Chicago, IL 60611, United States of America; 6 Department of Physics, Washington University in St. Louis, St. Louis, MO 63130, United States of America

**Keywords:** ecology, modeling, microbiology, complex systems, emergent behaviors, mechanistic microbial study, machine learning

## Abstract

In microbial systems, many biochemical functions arise from pathways encoded and executed at the community level. The collective nature of these functions complicates bottom-up efforts to determine each species’ contribution. Previous work has shown that regression over randomly sampled datasets of collective functions succeeds at predicting those functions. Building on this top-down idea, this paper asks whether regression can also reveal mechanistic insight into a cross-feeding relationship. For this, we propose extending the random sampling method to vary the growth environment, cultivating each community in the spent medium of another randomly constructed community. Using a model-based analysis, we illustrate that the mechanistic roles of species are structurally non-identifiable under the standard random sampling protocol, but become identifiable after the proposed modification, such that even a simple LASSO regression can recover them. More generally, our work suggests that the utility of machine-learning-based approaches can be greatly enhanced by a synergistic experimental design.

## 1. Introduction

Microbial communities, particularly those assembled naturally, are often very complex. The complex interactions among microbial strains often lead to collective functions that are absent when examining individual strains but emerge at the community level. Such functions underpin processes as varied as the degradation of environmental toxins [1], the breakdown of dietary fiber in the gut [2], and food fermentation [3], and they are the target of engineering efforts ranging from biomanufacturing on waste streams [4] to crop protection [5]. Surprisingly, these collective functions can sometimes be efficiently predicted with methods as simple as regression over presence-absence information of randomly-sampled species subsets [6, 7]. Here we call this sampling scheme the random subset protocol, as shown in figure 1(a).

**Figure 1.**
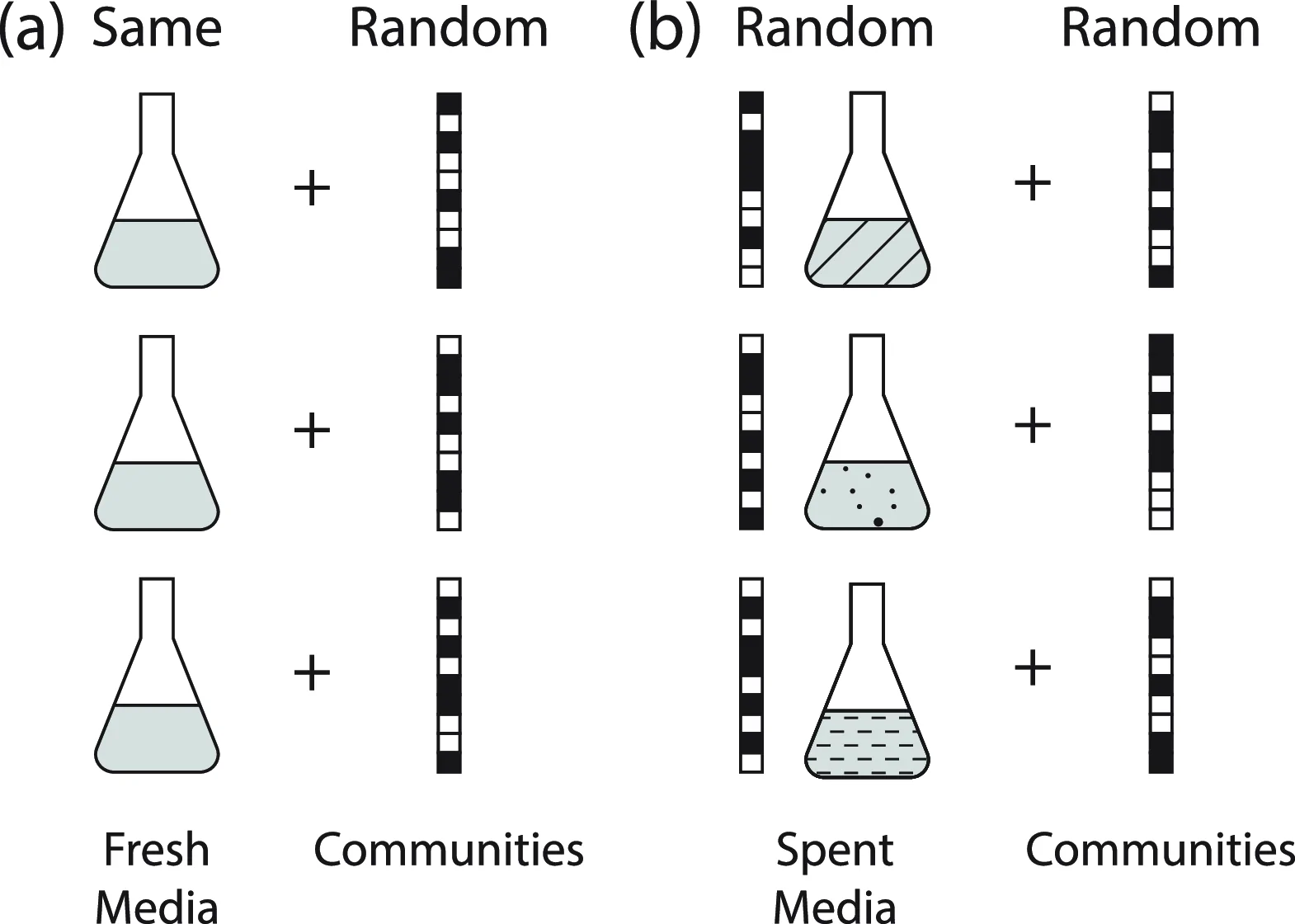
Experimental schemes for (a) the existing protocol of assaying random subsets of the species pool on the same fresh medium, and (b) our proposed random community-media pairing protocol, in which random communities are paired with random spent media. The binary vectors indicate species presence or absence in communities used to generate or pair with random spent media. The second protocol is expected to generate datasets with richer mechanistic information.

However, in biology our goal is often not just to predict (e.g. invasion resistance, or the rate of a metabolic process), but to understand something about the *mechanism* of its collective emergence in the community. While machine learning is increasingly seen as a powerful method to achieve the former, the latter—disentangling the mechanism—remains highly dependent on multiple omics-based analyses and distinctly more laborious [1–3, 8–11]. A recent study has succeeded in using random forest models to identify key species and interactions among 9 species with full combinatorial sampling at fixed community richness [12]. This success is exciting, but as the number of species increases, the process quickly becomes laborious again. Here, we ask whether simple machine learning methods, such as regression, can be leveraged to learn mechanistic information, without being exhaustive about sampling all possibilities.

In particular, we work in the context of a two-step cross-feeding pathway, and test both the random subset protocol and a modified protocol that we call random community-media pairing. As shown in figure 1(b), this protocol randomly pairs spent media with communities and measures a functional output for each pair. The modification is inspired by the long success of spent media analysis in disentangling cross-feeding relationships (for example, in gut microbiota [13], in biopolymer-degrading populations [14], and in soil microbiome [15]), as spent media carry information about which nutrients the cultured microbes consume and which byproducts they release. At its core, the new protocol amounts to two stages of random subset sampling. Falling costs of combinatorial community assembly [16] make protocols of this kind increasingly practical.

Using a model-based analysis, we show that the random subset protocol can fail to yield mechanistic information, while the modified protocol succeeds. Specifically, we find that random community-media pairing can assign species to the correct pathway steps and identify essential species, using a statistical method as simple as first-order LASSO regression. These results illustrate the value of random sampling in the mechanistic study of microbial systems, as well as the potential of modifying experimental protocols to enhance the extractability of mechanistic information by machine learning algorithms.

## 2. Results

### 2.1. A two-step pathway component model

To test the utility of random pairing protocol, we built a model to generate synthetic data. Because our goal here is to establish whether a change in experimental design can, in principle, make mechanistic information extractable at all, we deliberately work with synthetic data generated from a model with a known ground-truth mechanism. This lets us directly check whether an algorithm’s output matches the true underlying species roles—a comparison that is not possible with real communities, where the ground truth is generally unknown.

There are two requirements for the model. First, there should be at least two qualitatively distinct ways for a species to contribute to the function, so we can test the ability of our protocol to distinguish between them. Second, the synthetic function should be collective, in the following sense: Define a *functional community* as one for which the value of the functional readout exceeds some threshold (e.g. zero). Define a *minimal* functional community as one where all members are required: dropping any member makes the community non-functional. Now, let K denote the number of species in a typical minimal functional community (which need not be unique, due to functional redundancy between species). This K quantifies the collective nature of the function. If K is low (K = 1–2), then examining species individually or pairwise is sufficient to fully understand which species are contributing. The challenge arises when we are unable to reproduce some function in a low-complexity community, as is the case, e.g. for pathogen colonization resistance [17] and efficacious fecal microbiota transplantation [18]. This is the scenario where the traditional approaches struggle, and where randomized methods can be useful [6]. Thus, to test the random pairing protocol, our goal is to select a minimal model that satisfies these two requirements simultaneously.

To satisfy these two conditions, we adopted a model where a complex compound is synthesized through a two-step pathway, each requiring multiple species to run. Examples of such pathways include sugar fermentation in the gut microbiome [2, 19] and BPA degradation [1]. Conceptually, we consider a scenario where a complex compound (‘final compound’) is synthesized from another complex compound (‘precursor’), as shown in figure 2. In our model, both of these steps require cooperation of multiple species, but only the final compound concentration is measurable; the precursor is assumed to be unknown and not measurable. Our goal is to identify which species are participating in the precursor synthesis, and which species participate in the conversion of the precursor into the final compound.

**Figure 2.**
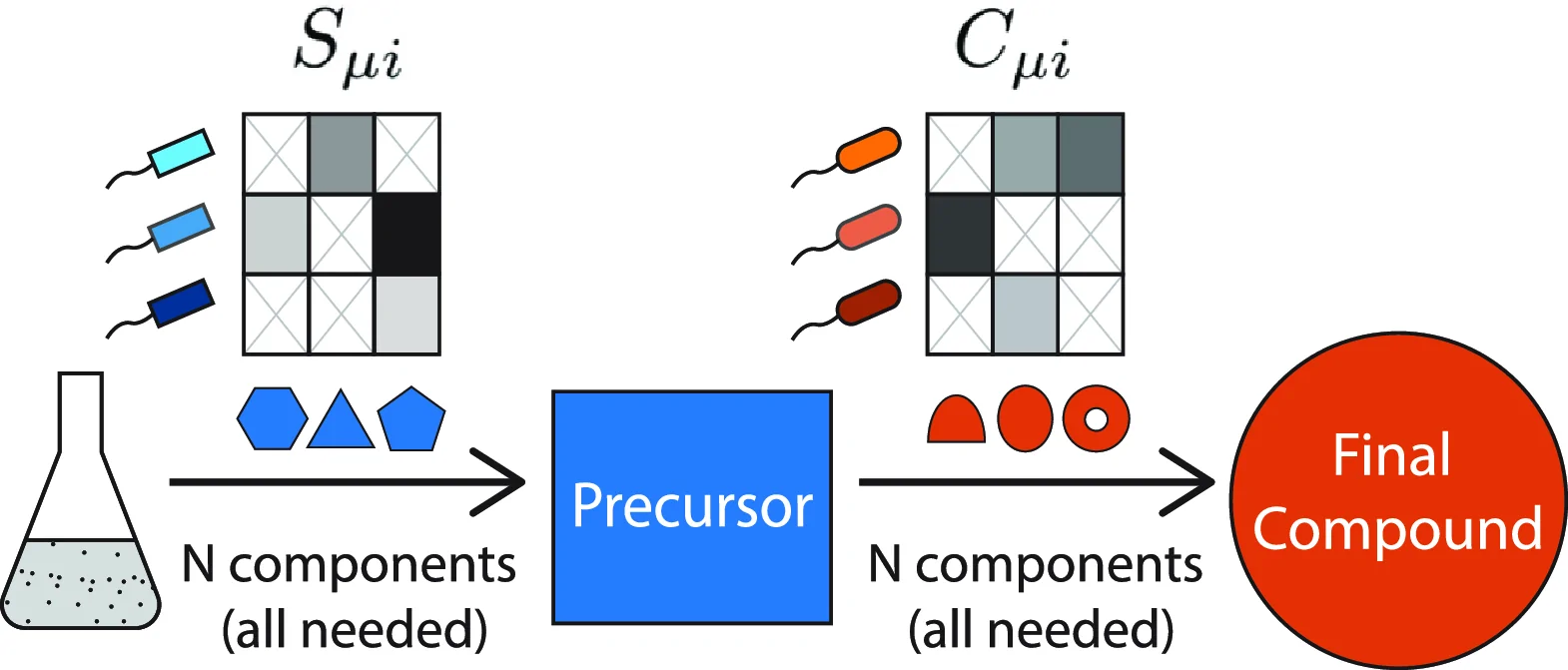
Two-step pathway component model as a minimal computational model with a clear mechanism to be recovered. Precursor is needed for the production of the final compound. For simplicity, species are divided into precursor makers (blue) and precursor converters (orange). The production of precursor and final compound each requires the presence of the complete set of N components of the respective pathway (for simplicity, N=10 in both cases). Each species can contribute to multiple components. $S_{\mu i}$ and $C_{\mu i}$ encode species’ contributions to precursor synthesis and precursor conversion, respectively. Synthetic data generated with this model were used to test the ability of regression to distinguish precursor makers from precursor converters.

We assume that precursor synthesis requires N pathway ‘components.’ Each species μ may contribute some amounts of components $i\in1\dots N$ of this precursor synthesis pathway, as characterized by a sparse matrix $S_{\mu i}$. Successful synthesis requires all N components to be present in the community.

Specifically, in the scenario of random subset protocol illustrated in figure 1(a), if $x_\mu \in \{0,1\}$ is the binary vector identifying which species were selected to be part of the community, we postulate the final precursor concentration to be:

\begin{align*} P = \mathrm{min}_i\,\left( \sum_\mu x_\mu S_{\mu i} \right).\end{align*}

In other words, synthesis is limited by the component with the lowest representation in the community. For simplicity, we do not explicitly model fresh medium, equivalently assuming that exactly the same fresh medium is used across all trials and that the composition of the cultured community determines the production of precursor.

Precursor conversion into final compound is implemented in a similar way: each species μ contributes components of a precursor conversion pathway, as described by a sparse matrix $C_{\mu i}$. The number of components of this pathway is also taken to be N for simplicity. The final compound concentration is given by:

\begin{align*} F = P \cdot \mathrm{min}_i\,\left( \sum_\mu x_\mu C_{\mu i} \right).\end{align*}

Here, P is the precursor concentration computed for this community as above. The final compound concentration F is proportional to the amount of precursor, and successful conversion requires all conversion pathway components to be present in the community. Equations (1) and (2) together describe the two-step pathway component model for the random subset protocol scenario in figure 1(a).

For the two-stage random community-media pairing protocol illustrated in figure 1(b), we use a similar set of equations. Specifically, we first create a library of spent media α (each defined by binary presence/absence vector of species, which we call $y_\mu^{(\alpha)}$), and then perform measurements of the final compound F created by a random subset of species $x_\mu^{(\alpha)}$ when placed in that spent medium $y_\mu^{(\alpha)}$. Any precursor and final compound are inherited through the medium:

\begin{align*} P_y &amp; = \min_i\,\left( \sum_\mu y_\mu S_{\mu i} \right)\end{align*}

\begin{align*} F_y &amp; = P_y \cdot \min_i\,\left( \sum_\mu y_\mu C_{\mu i} \right)\end{align*}

\begin{align*} P_x &amp; = P_y + \min_i\,\left( \sum_\mu x_\mu S_{\mu i} \right)\end{align*}

\begin{align*} F_x &amp; = F_y + P_x \cdot \min_i\,\left( \sum_\mu x_\mu C_{\mu i} \right)\end{align*}

Equations (3) and (4) describe the synthesis of precursor and final compound in the medium-constructing community, and are identical to equations (1) and (2). Equation (5) says that the precursor created by $x_\mu$ adds to the precursor already in the medium (created by $y_\mu$). We assume that the precursor-to-final-compound conversion rate is very small (F≪P), so that the conversion to final compound does not appreciably reduce the precursor concentration. Finally, equation (6) describes the conversion of precursor into final compound by community $x_\mu$, with the new synthesis being proportional to the full precursor concentration in the community (which includes any precursor inherited through the medium).

Note that in this model, we do not track the dynamics of species abundances. Instead, this is directly a model of a complex ‘functional landscape,’ defined as a mapping from either community composition $x_\mu$, as in the random subset protocol, or a pair of community composition and spent media label $(x_\mu, y_\mu)$, as in the random community-media pairing protocol, into a functional property (in this case, final compound concentration F) [6, 20–22]. The mapping we defined is highly non-linear due to the minimum function, and since the matrices S and C are sparse, the production of precursor and the conversion to final compound usually each requires multiple species to be present simultaneously (higher-order interactions). The precursor synthesis matrix S and precursor conversion matrix C are the mechanistic ‘ground truth’ that characterizes how a given species is contributing to the community-level function F (final compound concentration).

For simplicity, here we first assume that each species may contribute either precursor synthesis components or precursor conversion components, but not both. (If row μ of matrix C has a non-zero entry, the corresponding row in matrix S is all zeros, and vice versa. The zero half of $S_{\mu i}$ corresponding to precursor converters and the zero half of $C_{\mu i}$ corresponding to precursor makers are omitted in figure 2 for simplicity.) This assumption turns the problem of distinguishing species’ contribution types into a binary classification problem, which allows for an easier definition of a performance metric. Specifically, we evaluate performance using the Matthews correlation coefficient (MCC), calculated between the true classification of species as a precursor-maker or a precursor converter and the algorithm’s best guess. The MCC is a standard method of evaluating the performance of binary classifiers [23].

### 2.2. Random subset protocol is insufficient for mechanistic species classification

Our model implements a minimal scenario where being able to predict F does not, on its own, answer the mechanistic question of how each species contributes to the function. Indeed, if we observe that adding a species μ to some community increases the final compound concentration F, this could be for either of two reasons: μ might contribute to precursor synthesis, or might improve its conversion into final compound. In this section, we show that using random subset protocol poses a challenge to distinguish between the two types of contributions.

We first check that our pathway component model is an example of a scenario where linear regression succeeds at prediction. We define richness as the typical number of species present in a randomly assembled community. We also introduce density, which refers to the probability that a precursor maker is capable of contributing to a given precursor-production component (so named because it relates to the fraction of nonzero entries in the pathway matrix). For simplicity, we use the same probability for a precursor converter contributing to a precursor-conversion component. A density of 1 corresponds to a fully redundant system in which every precursor maker can independently produce the precursor, and every precursor converter can independently carry out the conversion. As density decreases, production of the final compound becomes increasingly dependent on collective contributions from multiple species. For each pair of richness and density, we fit LASSO-regularized linear regression with 10-fold cross validation [24, 25] on a subset of the simulated trials and evaluated prediction performance on the held-out remainder, repeating the procedure over many independent realizations of the ground-truth matrices S and C (see Materials and Methods, section 4.1, for details). Figure 3(a) reports the mean of the prediction performance (measured in terms of the Pearson correlation coefficient between the predicted F and the true F). The panel shows that LASSO regression succeeds at prediction over a wide range of parameter space (the decrease in performance in the lower-left region is expected, as communities sampled are often non-functional). Hence, the model is within the desired scenario where regression is capable of prediction.

**Figure 3.**
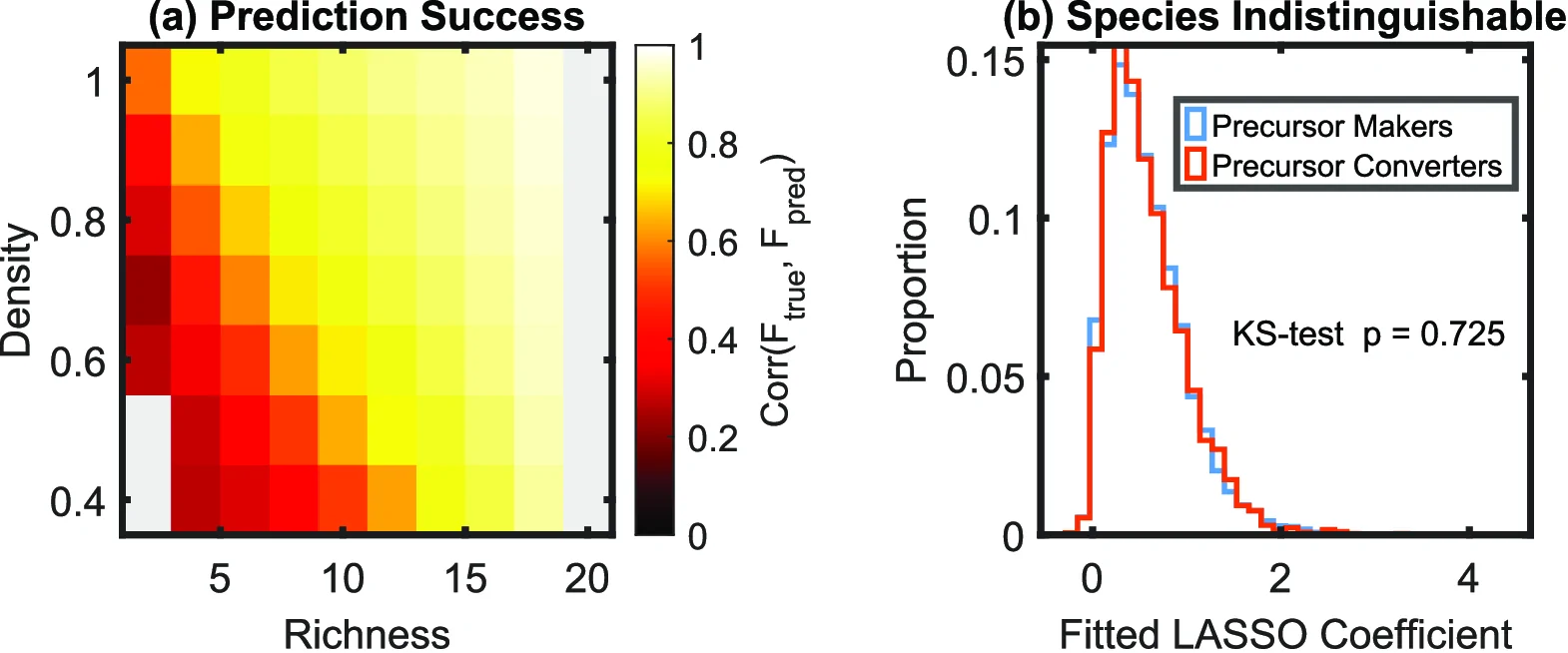
Sufficient for functional prediction, the existing protocol is insufficient for mechanistic species classification. (a) Simple regression is sufficient for function prediction. Despite the complexity of collective microbial functions, previous research shows that simple regression over presence-absence information of random communities and their respective functional readouts is sufficient for function prediction. As shown, our model operates in the parameter regime where simple LASSO regression succeeds at function prediction, consistent with prior empirical studies. (Density controls the probability of a species being able to produce a component. Richness refers to the typical number of species drawn from the species pool into a community. 20 species in the pool. 500 trials for each dataset, 500 datasets for each parameter pair. Dataset training-testing split is 90–10.) (b) Despite the success in prediction, random community data does not readily provide mechanistic information about how species contribute to the function. Statistically, the distributions of the fitted coefficients for different types of contributors are indistinguishable (KS-test: p = 0.725; see figure S1 for more analysis). This indistinguishability persists even when using more advanced algorithms and incorporating additional prior information (see Materials and Methods, section 4.3, and figure S2, S3).

However, for random subset datasets, while simple LASSO regression already succeeds at prediction, such datasets are not very informative about how species contribute to the final compound concentration F. Indeed, the distributions of the LASSO-fitted coefficients for the two types of species are statistically indistinguishable (see figure 3(b), S1). This is still the case if we switch to a more advanced algorithm based on random forest regression (RFR) (figure S2). Even when we recast the problem as a supervised learning task, providing the model with prior knowledge of half of the species’ identities, it still fails to classify the remaining unknown species (figure S3).

The reason for this consistent failure is straightforward: in our model, with F as the only observable, role assignment is demonstrably impossible. Indeed, the observable

\begin{align*} F = \min_i\left(\sum_\mu x_\mu S_{\mu i}\right) \min_i\left(\sum_\mu x_\mu C_{\mu i}\right)\end{align*}

is a product of two factors of identical form, and is therefore invariant under the exchange S↔C. Thus, for every community $\vec{x}$, any ground truth (S,C) produces exactly the same value of F as the swapped ground truth $(S^{^{\prime}},C^{^{\prime}})$, where $S^{^{\prime}} = C$ and $C^{^{\prime}} = S$, so that every precursor maker is instead a precursor converter and vice versa. Therefore, no dataset generated under this protocol can distinguish the two, however many communities are assayed. This means that S and C, i.e. role assignment, are structurally unidentifiable [26]. We ask whether an alternative random sampling scheme could break this limitation.

### 2.3. Random community-media pairing succeeds at extracting mechanistic information

The alternative experimental design we propose is the random community-media pairing protocol, as illustrated in figure 1(b). We generated synthetic datasets with the new protocol, again based on the two-step pathway component model. Each data point in such new datasets consists of two binary vectors, $\vec{y}$ and $\vec{x}$, and a scalar $F_x$, the level of the final compound at the end of each trial. As mentioned before, we assume that $F_x$ is the only quantity measured in the experiment. Mathematically, this protocol makes $F_x$ depend asymmetrically on S and C and breaks the non-identifiability problem described above (for details, see Materials and Methods, section 4.4).

We then empirically illustrate the usefulness of the random community-media pairing protocol using simple LASSO regression. Over each dataset (equivalent to one set of experiment), we trained LASSO regression with 10-fold cross validation, and LASSO regression returned two coefficients for each species, α and β, with α corresponding to $\vec{x}$ and β corresponding to $\vec{y}$. As figure 4(a) shows, $(\alpha,\beta)$’s tend to split on the two sides of the 1–1 line, with the splitting highly correlated with the types of the corresponding species. Inspired by this observation, we configured a classifier: for each species μ, if $\beta_\mu > \alpha_\mu$, then μ is classified as a precursor maker; conversely, if $\alpha_\mu > \beta_\mu$, then μ is classified as a precursor converter. We then checked the performance of this simple LASSO-based classifier in extracting mechanistic information about the collective function. If $\beta_\mu = \alpha_\mu$, we label a species as ‘unclassified’ and treat this as a classification error for conservative evaluation of classification performance (see Materials and Methods, section 4.5 for how such ties can sometimes be resolved).

**Figure 4.**
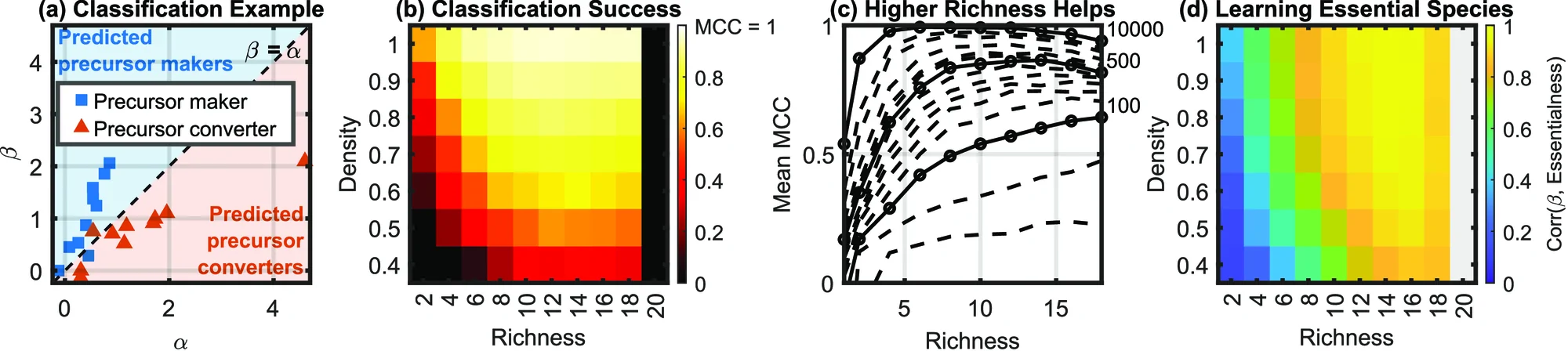
Random community-media pairing succeeds at extracting mechanistic information. (a) Illustration of classification process using LASSO-fitted coefficients over random community-media pairing datasets. Each species has two fitted coefficients: α and β, corresponding to the random communities stage and the random media stage, respectively. If $\beta > \alpha$, the species is classified as a precursor maker; conversely, if $\alpha > \beta$, the species is classified as a precursor converter. Rest of this figure applies this classifier. (b) With random community-media pairing, mechanistic information was easily extracted. LASSO-based classification outputs were compared with ground truth, performance measured with Matthews correlation coefficient (MCC). 500 trials for each dataset, 500 datasets for each parameter pair. Plotted are means over the 500 datasets. Low performance at the lower-left corner is expected, as the assembled communities are usually non-functional (figure S4). Simpleness of this LASSO-based classifier highlights the mechanism-revealing strength of random community-media pairing. (c) Including more species in communities is helpful. Shown are performance curves with different trial numbers for density = 0.8. Bold curves correspond to trial number =[100,500,10000]. Data points cover richness =[1,2,4,6,8,10,12,14,16,18]. From bottom to top, curves shown correspond to trial number $=\;\lbrack30,\;50,\;100,\;150,\;200,\;250,\;300,\;400,\;500,\;600,\;700,\;1000,\;2000,\;3000,\;10\:000\rbrack$. Increasing community richness significantly increased classification performance, especially for low-trial-number scenarios. (d) Random community-media pairing helps extract additional mechanistic information about essential species. Species essentialness is defined by the likelihood of each component being the bottleneck that limits the production of either the precursor or final compound, along with species’ abilities to produce the component (see Materials and Methods, section 4.6). As shown, for predicted precursor makers, their essentialness values correlate strongly with their fitted β values. The same holds true for predicted precursor converters (showing a high correlation with fitted α’s), and both correlations are statistically significant (see figure S8). Such high correlations mean that random community-media pairing enables further mechanistic understanding of the species pools.

As shown in figure 4(b), the classification has a wide success range in the density-richness parameter space. Here, richness refers to the typical number of species used to construct a spent medium and the typical number of species in a subset community (same richness for simplicity). The sub-optimal performance at the lower-left corner is understandable, as most communities assembled in this parameter regime are non-functional (figure S4). As expected, classification performance generally decreases with density. Lower density corresponds to higher collectivity of function, as typically more species are needed to form a functional community (see figure S5 for details). However, this performance drop can be compensated by increasing community richness (which yields more high-functioning examples). The wide range of classification success shows that role assignment becomes identifiable with the new protocol. Even when we remove the assumption of binary species roles, LASSO regression continues to extract information about the relative contribution of each species to the two steps (figure S6). While the number of trials needed to reach decent classification performance is typically high (figure 4(c) and figure S7), this is not a concern here, as our purpose is to illustrate that a change in random sampling can render mechanistic information identifiable. In practical cases, one can choose more advanced algorithms catering to their specific systems of interest.

Figure 4(c) further highlights the general increase of classification performance with richness. In other words, the protocol presented here succeeds not *despite* high richness of trial communities, but rather *because* of it, as richness generally improves performance. (The slight non-monotonicity at high richness observed for very large trial numbers is expected: for any finite species pool, once the richness reaches half of the total number of species, increasing it further will make the assembled communities increasingly alike; for instance, there is only one possible community when richness equals the total number of species, and therefore corresponding classification performance collapses, as shown in the rightmost column of figure 4(b)). Although our minimal model is not meant as a faithful representation of any particular microbial system, these observations indicate the promise of randomized sampling of large subsets of species pool in the mechanistic study of collective microbial functions.

In addition to classifying species, the LASSO-based classifier over data generated by random community-media pairing also extracts mechanistic information about the ‘essentialness’ of each species for the collective function. In our pathway component model, the production of precursor is limited by the least available component in the system; the same holds true for the conversion from precursor to final compound. We call such components bottleneck components. To assess whether the classifier can learn about species essential to the collective function, we quantify the ‘essentialness’ of a species as the sum of the species’ ability to generate each component, weighted by the likelihood of that component being the bottleneck component (see Materials and Methods, section 4.6, for details). The two sets of statistics produced by the classifier, β’s and α’s, are found to strongly correlate with the essentialness of predicted precursor makers and predicted precursor converters, respectively (figure 4(d), S8). It is noteworthy that, for high richness, the two statistics remain highly correlated with species essentialness even when the classification performance is sub-optimal (e.g. density = 0.4) (figure 4(b), (d), S8). Thus, at least in our model, the protocol succeeds at identifying species essential to the collective function.

## 3. Discussion

Together, these results provide a proof of principle that the merit of random sampling methods for studying collective microbial functions may be extended beyond approximate predictions, and towards extracting mechanistic information. Specifically, we show *in silico* that a regression method over randomized data can reveal mechanistic insights about a collective microbial function: We built a minimal two-step pathway model that generated synthetic data following the random pairing protocol, which randomly pairs spent media with species subsets; we showed that LASSO regression over this dataset is enough to classify species by the way of their contribution to the collective function—either contributing to the generation of the precursor or helping to convert the precursor to the final compound; the LASSO-fitted coefficients also extract additional mechanistic information about essential species that are most likely to cause bottlenecks in either the production of the precursor or the production of the final compound. These results demonstrate how a shift in experimental design—from random subset protocol to random community-media pairing—can enable the extraction of mechanistic information.

In biological studies seeking mechanistic insight, machine learning has been applied in two principal ways. The first one is to develop algorithms whose output would be more mechanistically interpretable [27–29] (figure 5(a)). The second approach, known as active learning [30–32] (figure 5(b)), is to iteratively use machine learning to predict which additional data points are the most informative and hence should be measured next. Complementing these approaches, our results highlight the potential of integrating machine learning at the level of a synergistic experiment design (figure 5(c)). Specifically, in our analysis, even an extremely simple algorithm (linear regression) can reveal much more insight with an appropriately modified data collection protocol. Given the highly abstract nature of our model, this strategy of ML-synergistic experimental design could perhaps also be useful for other collective biological systems.

**Figure 5.**
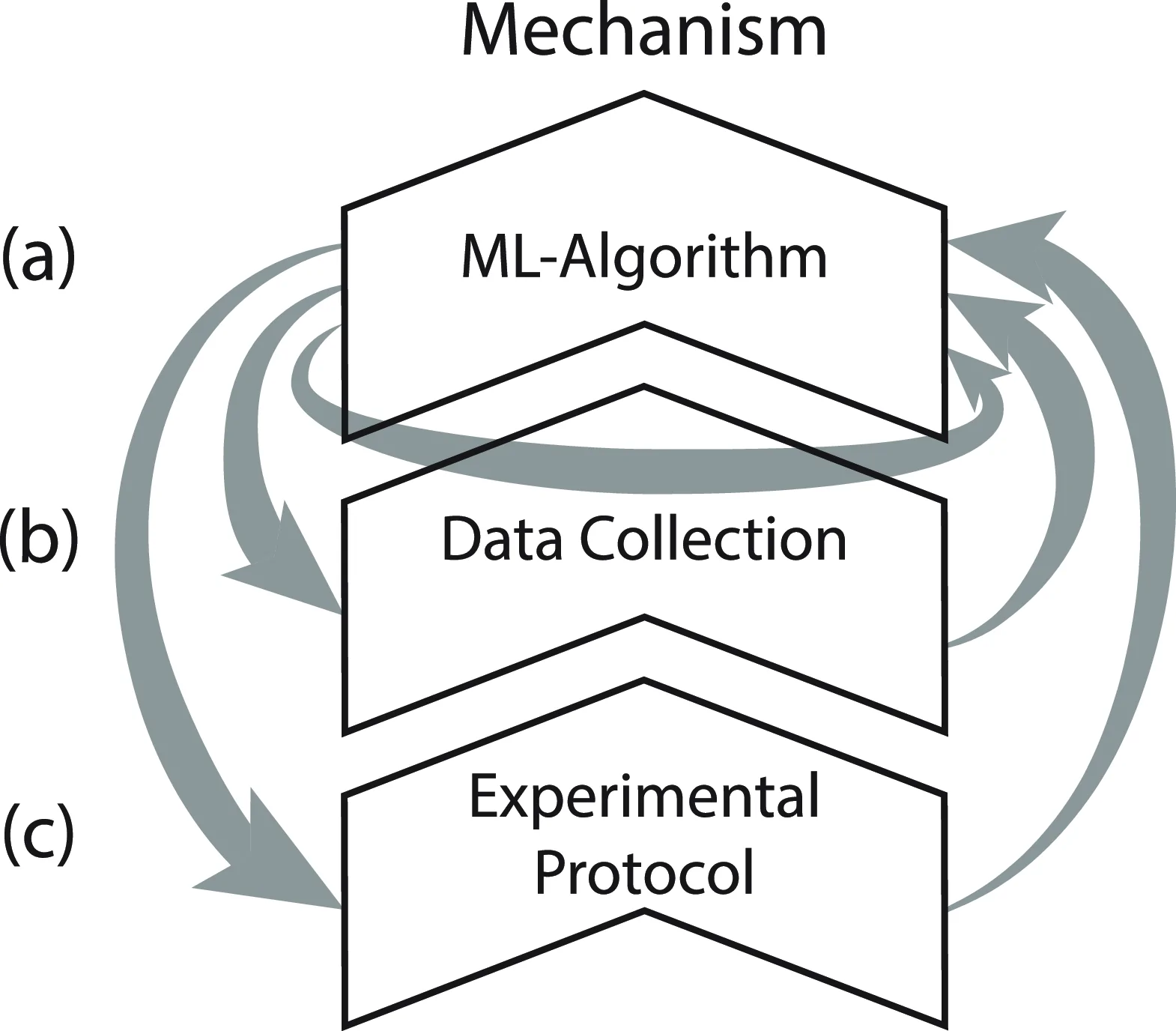
Tiers of machine learning (ML) integration into studies of collective microbial function. The dominant two approaches of harnessing ML approaches for deriving mechanistic biological insight are (a) improving the interpretability of ML algorithms and (b) using ML to guide data collection through active learning, which predicts the most informative measurements to measure next. (c) Our results highlight a complementary third strategy: ‘ML-synergistic experimental design.’ In the example we presented, the two-step experimental design allows even simple regression models to recover mechanistic information about the collective function.

In real-world experiments, there are two easily available improvements for extracting mechanistic information. First, after assembling a spent medium, it is natural to measure its composition (for example, as used in [13]). How this spent medium differs from the fresh medium yields additional information about the species being used to make the spent medium. Another improvement is that one can use an algorithm more advanced than regression. With LASSO regression, the number of trials needed to reach decent classification performance is typically large (figure 4(c) and S7). In this paper, we were less concerned by efficiency of inference, but intentionally limited our inference to the minimal ‘one functional measurement per pairing’ and simple LASSO regression to illustrate the core idea.

This study has two caveats. First, in our model, we assumed fixed, non-linear interactions among the microbial species. Realistic metabolic pathways may deviate from this assumption. Second, while we observed that increasing richness can be helpful for learning mechanistic information, we cannot offer a rule-of-thumb on choosing the optimal richness, which would likely be highly dependent on the system. Overall, more computational and experimental work regarding specific systems are needed to establish the practicality of the proposed protocol.

## 4. Materials and Methods

### 4.1. Simulation details

We worked with microbial pools of 20 species, consisting of 10 precursor makers and 10 precursor converters. Each pool is defined by a pair of pathway–component matrices, S and C. Density controls the percentage of non-zero entries in S and C. The value of each non-zero entry is drawn from an exponential distribution with mean 1.

For the random subset protocol, we generated 500 independent ground-truth (S,C) pairs for each pair of richness and density. For each such pair, we generated 500 independent community subsets $\vec{x}$ and their corresponding final compound levels F; we then fit LASSO regression (10-fold cross-validated) on 450 of these trials to predict F from $\vec{x}$, and evaluated prediction performance on the remaining held-out 50 trials. Averaging over the 500 (S,C) pairs then yielded stable estimates for each (richness, density) combination.

For the random community-media pairing protocol, we likewise generated 500 independent ground-truth (S,C) pairs for each pair of richness and density. For each such pair, we generated 500 independent trials—each consisting of a community-medium pair $(\vec{x},\vec{y})$ and its resulting F—and fit LASSO regression (10-fold cross-validated) to predict F from $(\vec{x},\vec{y})$, yielding the coefficient vectors α and β. Unlike the random subset protocol, the full set of 500 trials was used for fitting, with no held-out test split, since classification performance here is assessed directly from the fitted coefficients rather than from predictive accuracy. Averaging over the 500 (S,C) pairs again yielded stable estimates for each (richness, density) combination.

All simulations were performed in MATLAB (Mathworks, Inc.). The associated code, simulation data and scripts to reproduce all figures in this work are publicly available [33].

### 4.2. MCC

This paper uses MCC to evaluate classification performances. MCC is a robust single-value classification metric for balanced datasets, in which positive and negative cases hold equal importance [23]. MCC can be expressed as:

\begin{equation*} \mathrm{MCC} = \frac{TP \cdot TN - FP \cdot FN}{\sqrt{\left(TP + FP\right)\left(TP + FN\right)\left(TN + FP\right)\left(TN + FN\right)}},\end{equation*}

where TP, TN, FP, and FN correspond to true positives, true negatives, false positives, and false negatives, respectively. $\mathrm{MCC} = 1$ means perfect classification; $\mathrm{MCC} = -1$ means perfect mis-classification; and $\mathrm{MCC} = 0$ corresponds to random classification. We arbitrarily regarded being precursor makers as the positive cases, and being precursor converters the negative. This choice has no impact on the evaluation, as $\mathrm{MCC}$ is symmetrical with respect to positive and negative cases. Additionally, we treated non-classification as false classification for conservative evaluations of $\mathrm{MCC}$’s.

### 4.3. RFR classification attempts with random subset datasets

We also used classifiers based on RFR over the random subset datasets.

**Unsupervised** — Given the classification failure of RFR on random subset dataset, we further checked if a more sophisticated unsupervised algorithm using RFR will be able to succeed. We first trained RFR for function prediction (performance shown in figure S2(a)). Then, to leverage the intuition that precursor makers and precursor converters contribute to the collective function differently, we used the trained RFR model to construct species contribution profile vectors, which we define as the RFR-predicted effect of perturbing presence/absence of the respective species across all trials. K-means over species contribution profile vectors then divided species into two clusters. Each cluster was leniently labeled with the true majority identity of its constituents; species were classified with cluster labels. Figure S2(b) records the consistent failure of this method at classification.

**Supervised** — We further lessen the difficulty of the task by allowing prior knowledge about the identities of half of the species pool (5 known precursor makers and 5 known precursor converters) and turning the task into a supervised classification problem. Same method as aforementioned was used to generate the contribution profile vectors. Linear SVM and logistic regression were trained on the contribution profile vectors of the 10 known species and then used to classify the 10 unknown species. Classification performance was then evaluated with regard to the 10 unknown species. Figure S3 shows the continued failure of classification.

These failures with more advanced classifiers and additional information demonstrate the underdesirability of random subset protocol for extracting mechanistic information.

### 4.4. Identifiability under random community-media pairing

Here we show analytically how the random community-media pairing protocol breaks the degeneracy that the random subset protocol exhibits under the exchange of S and C.

Denote

\begin{align*} a = \min_i\left(\sum_\mu y_\mu S_{\mu i}\right), \qquad b = \min_i\left(\sum_\mu y_\mu C_{\mu i}\right)\end{align*}

for the community that constructs the medium, and

\begin{align*} c = \min_i\left(\sum_\mu x_\mu S_{\mu i}\right), \qquad d = \min_i\left(\sum_\mu x_\mu C_{\mu i}\right)\end{align*}

for the community assayed in that spent medium. Equations (3)–(6) then give the true final compound concentration as

\begin{align*} F_x = ab + \left(a+c\right)\,d .\end{align*}

The exchange S↔C sends this quantity to $F_x^{^{\prime}} = ab + (b+d)\,c$, and the difference between the two is

\begin{align*} F_x - \left. F_x^{^{\prime}} \right|_{S \leftrightarrow C} = ad - bc ,\end{align*}

which is generically nonzero. The observable of the paired protocol therefore separates the two ground-truth role assignments that random subset data cannot. It is worth noting that the difference ad−bc vanishes when $\vec{x} = \vec{y}$ (this is equivalent to assaying a community in its own spent medium), and the two role assignments yield the same observable again. This is not a concern for the random community-media pairing protocol, as it only constitutes one of the many possible communities.

### 4.5. LASSO-based classifier

For each species μ in the binary case, we classify its contribution type using the sign of $\beta_\mu - \alpha_\mu$, where $\alpha_\mu$ and $\beta_\mu$ are the LASSO coefficients associated with the community-assembly stage ($\vec{x}$) and the spent-medium-generating stage ($\vec{y}$), respectively, taken at the value of λ that minimizes the cross-validated mean squared error ($\lambda_{\min}$). Species with $\beta_\mu - \alpha_\mu > 0$ are classified as precursor makers, and those with $\beta_\mu - \alpha_\mu < 0$ as precursor converters.

A tie ($\beta_\mu = \alpha_\mu$) occurs when both coefficients take the same value at $\lambda_{\min}$, which in practice arises almost exclusively because the $L^1$ penalty has shrunk both to exactly zero. Rather than immediately labeling such a species as unclassified, we first attempt to resolve the tie using the rest of the LASSO regularization path: starting from $\lambda_{\min}$, we step through the fitted λ sequence toward smaller λ (i.e. toward the less-regularized, denser end of the path), and at each step re-evaluate the sign of $\beta_\mu - \alpha_\mu$ for any species still unresolved. As soon as a species’ sign becomes nonzero at some λ along this direction, it is assigned the corresponding label. The procedure terminates once every species has been classified, or once the least-regularized end of the path is reached. Any species that remains tied throughout is finally labeled ‘unclassified’ and treated as a classification error, for conservative evaluation of classifier performance.

### 4.6. Bottleneck components and species essentialness

Bottleneck components refer to the components i that limit either the production of precursor or the conversion of precursor to the final compound. In other words, bottleneck components are the components at which the minimums in equations (3)–(6) are taken. These bottleneck events are recorded separately for the random media phase and the random community phase and then pooled together. A small Gaussian noise ($\varepsilon = 10^{-10}$) is intentionally added when determining the bottleneck component in order to break potential degeneracies when two or more components have identical amounts available; without this perturbation, min() in MATLAB would always return the earliest component in the list. This noise is used only for selecting the bottleneck index and is not included in the calculation of precursor production or conversion to the final compound. Additionally, a bottleneck component on the conversion step is only returned when precursor is present in the system.

For a component i in the precursor synthesis matrix S, its bottleneck likelihood $b_S(i)$ is defined as the number of times component i was identified as a bottleneck, normalized by the total number of bottleneck events observed for that pathway step. $b_C(i)$ is defined analogously for components in the precursor conversion matrix C. For each pair of pathway matrices S and C, we simulate $10{\,}000$ additional trials to estimate these likelihoods, so that the estimates are independent from the trials used to train the LASSO regression.

Species essentialness is then defined using these bottleneck likelihoods. The essentialness of species μ is defined as

\begin{equation*} E_\mu = \sum_{i} S_{\mu i}\, b_S\left(i\right) \;+\; \sum_{i} C_{\mu i}\, b_C\left(i\right).\end{equation*}

Thus, a species is regarded as more essential if it contributes strongly to components that are frequently limiting in the system.

### 4.7. Non-binary role assignment

To relax the assumption that each species contributes to one pathway step and not the other, the matrices S and C were first generated exactly as in section 4.1, and each species μ was then assigned a fraction $p_\mu$. Of that species’ $n_\mu$ non-zero pathway components, $\mathrm{round}(p_\mu n_\mu)$ were chosen at random and moved out of its home matrix into randomly chosen components of the other matrix that the species did not already occupy, with the magnitude of each entry preserved. Entries are relocated rather than duplicated, so both the number of components a species contributes to and the magnitudes of those contributions are unchanged; only their allocation between the two pathway steps differs. Thus $p_\mu = 0$ leaves a species pure, $p_\mu = 1$ turns it into a pure member of the opposite type, and intermediate values give species contributing to both steps.

The values of $p_\mu$ were taken to be equally spaced on [0,1] within each half of the species pool, so that the species which are precursor makers in the binary case and those which are converters each span the full range from pure to completely reassigned. Simulations used the following parameters: 20 species, 10 components per pathway step, original density 0.8, richness 10, and 500 pairings per dataset, pooled over 500 replicate datasets.

A species’ relative involvement in the two pathway steps is quantified by

\begin{align*} \Delta_\mu = \frac{\sum_i S_{\mu i} - \sum_i C_{\mu i}} {\sum_i S_{\mu i} + \sum_i C_{\mu i}},\end{align*}

so that $\Delta_\mu = 1$ for a pure precursor maker and $\Delta_\mu = -1$ for a pure precursor converter. For each species we recorded $\Delta_\mu$ together with the angle $\phi_\mu = \tan^{-1}(\beta_\mu / \alpha_\mu)$ formed by its two LASSO-fitted coefficients. Species for which LASSO returned $\alpha_\mu = \beta_\mu = 0$ have no defined angle and were discarded. Figure S6 restricts the display to $\phi_\mu \in [0^\circ, 90^\circ]$, equivalently $\alpha_\mu {\unicode{x2A7E}} 0$ and $\beta_\mu {\unicode{x2A7E}} 0$, which retains 94.9% of species; the Pearson and Spearman correlations reported are then computed on that subset.

## Data Availability

The data that support the findings of this study are openly available at the following URL/DOI: https://doi.org/10.17632/8f6gnsrw2y.2 [33]. Supplementary data 1 available at https://doi.org/10.1088/1478-3975/aeb3c4/data1.
